# Soluble Triggering Receptors Expressed on Myeloid Cells (sTREM) in Acute Ischemic Stroke: A Potential Pathway of sTREM-1 and sTREM-2 Associated with Disease Severity

**DOI:** 10.3390/ijms25147611

**Published:** 2024-07-11

**Authors:** Greta Salafia, Angelica Carandina, Roberto Maria Sacco, Evelyn Ferri, Nicola Montano, Beatrice Arosio, Eleonora Tobaldini

**Affiliations:** 1 Department of Clinical Sciences and Community Health, Dipartimento di Eccellenza 2023–2027, University of Milan, 20122 Milan, Italy; greta.salafia@unimi.it (G.S.); angelica.carandina@unimi.it (A.C.); nicola.montano@unimi.it (N.M.); beatrice.arosio@unimi.it (B.A.); 2 Emergency Department, Fondazione IRCCS Ca’ Granda Ospedale Maggiore Policlinico, 20122 Milan, Italy; roberto.sacco@policlinico.mi.it; 3 Geriatric Unit, Fondazione IRCCS Ca’ Granda Ospedale Maggiore Policlinico, 20122 Milan, Italy; evelyn.ferri@policlinico.mi.it; 4 Department of Internal Medicine, Fondazione IRCCS Ca’ Granda Ospedale Maggiore Policlinico, 20122 Milan, Italy

**Keywords:** ischemic stroke, neuroinflammation, soluble TREM-1, soluble TREM-2

## Abstract

In 2022, stroke emerged as the most significant cerebrovascular disorder globally, causing 6.55 million deaths. Microglia, crucial for CNS preservation, can exacerbate brain damage in ischemic stroke by triggering neuroinflammation. This process is mediated by receptors on microglia, triggering receptors expressed on myeloid cells (TREM-1 and TREM-2), which have contrasting roles in neuroinflammation. In this study, we recruited 38 patients within 4.5 h from the onset of ischemic stroke. The degree of severity was evaluated by means of the National Institutes of Health Stroke Scale (NIHSS) at admission (T0) and after one week of ischemic events (TW) and the Modified Rankin Scale (mRS) at three months. The plasma concentration of TREMs (sTREM) was analyzed by next-generation ELISA at T0 and TW. The sTREM-1 concentrations at T0 were associated with mRS, while the sTREM-2 concentrations at T0 were associated with both the NIHSS at T0 and the mRS. A strong correlation between sTREM-1 and sTREM-2 was observed, suggesting a dependent modulation of the levels. This study provides insights into the potential pathway of TREM-1 and TREM-2 as a future biomarker for stratifying high-risk patients with ischemic stroke.

## 1. Introduction

Stroke stands out as the most significant and devastating clinical manifestation among all cerebrovascular disorders [1]. The most recent statistics estimated that stroke caused 6.55 million deaths (84.69 per 100,000) in 2022, making it the second leading cause of death after ischemic heart disease, with an incidence of new cases of around 7.6 million (94.51 per 100,000) [2].

Ischemic stroke is caused by the occlusion of a cerebral artery, resulting in a reduction in blood supply, and, consequently, oxygen and nutrients, which leads to alterations in the functionality of brain cells.

In various cerebrovascular diseases, microglia play a fundamental role in the preservation of the Central Nervous System (CNS) by eliminating necrotic tissue and apoptotic cells [3].

In ischemic stroke, microglia quickly activate and accumulate at the infarction site, demonstrating the ability to phagocytize vital cells instead of just eliminating cellular debris, thereby exacerbating brain damage or disrupting normal pathophysiological mechanisms, as observed in the penumbra of stroke or neurodegenerative diseases [4].

Neuroinflammation, guided by microglial cells, is a key component in the ischemic cascade that results in cell damage, disability, and poor prognosis after cerebral ischemia due to the chronic release of high levels of pro-inflammatory cytokines and chemokines [5,6].

Triggering receptors expressed on myeloid cells (TREM-1 and TREM-2) are a family of receptors involved in the immune system expressed on a variety of innate cells of the myeloid lineage, including microglia [7].

The shedding of the receptor ectodomain by secretases produces soluble forms of the receptors, sTREM-1 and sTREM-2, detectable in the peripheral blood and cerebrospinal fluid (CSF) [8].

The mechanisms of the release of soluble forms remain uncertain, and it is hypothesized that may function as a regulatory mechanism, enhancing or decreasing the activity of TREM receptors. Soluble TREMs can act as natural antagonists of membrane receptors [9,10].

TREM-1 was initially described as a useful pro-inflammatory prognostic biomarker in sepsis, and it has also been described in different neurological diseases, such as stroke, Alzheimer’s disease, and Parkinson’s [11,12].

Regarding acute ischemic stroke (AIS), studies in animals have found that pharmacological induction of ischemia promotes TREM-1 receptor activation, which induces the production of pro-inflammatory cytokines and chemokines in microglia, suggesting a critical role in post-ischemic neuroinflammation [13].

In humans experiencing acute ischemic stroke, the serum levels of sTREM-1 exhibited a strong correlation with the concentrations of human S100 calcium-binding protein B, along with the serum levels of pro-inflammatory cytokines, such as TNF-α, IL1β, IL6, IL8, and IFN-γ, as well as the stroke volume and the NIHSS [14].

On the other hand, TREM-2 appears to play a critical role in maintaining tissue homeostasis by promoting the removal of cellular debris through phagocytosis and modulating inflammation, particularly neuroinflammation. However, the role of TREM-2 in ischemic stroke remains uncertain.

Studies in TREM-2 knockout mice show reduced quantities of activated microglia and phagocytes, leading to impaired clearance of apoptotic or necrotic cells and hindering ischemic tissue elimination [15].

By contrast, elevated levels of soluble TREM-2 (sTREM-2) are associated with higher inflammatory markers (e.g., hsCRP) [16].

As several studies have reported that sTREM-2 appears to be involved in different pathways, such as cardiovascular events and the immune response after AIS, it makes sTREM-2 an interesting biomarker to investigate [17,18].

As data regarding the assessment of soluble TREMs in patients with ischemic stroke are limited in the literature and controversial for sTREM-2, the aim of our study was to investigate the role of sTREM-2 and its interaction with sTREM-1 during the hyperacute post-ischemic phase, analyzing their plasma concentrations in relation to stroke severity and outcomes at three months post-acute event.

## 2. Results

A total of 38 patients with hyperacute stroke (within 4.5 h from the beginning of symptoms), 23 males and 15 females, were studied. Briefly, the average age of the participants was 72.9 years. The percentage of smokers was 23.7%.

Additionally, 23.7% of the patients had a history of previous stroke or transient ischemic attack (TIA).

A total of 63.2% of the patients had hypertension, 15.8% had diabetes, and 31.6% experienced prior episodes of atrial fibrillation. (Table 1).

Based on the Trial of Org 10,172 in the Acute Stroke Treatment (TOAST) classification, a higher percentage of people was affected by cardioembolic (50.0%), atherothrombotic (13.4%), lacunar (5.3%), and undetermined etiology (31.6%) stroke.

Both the hemispheres (right and left) were involved in equal parts (47.3%), with two exceptions of bilateral involvement (5.26%).

The vascular territory involved was anterior in the highest percentage of people (81.58%).

Categorizing the patients according to the TOAST classification, we did not show any difference between the groups (Supplementary Table S1). Regarding the vascular territory, there was a slight significance between anterior and posterior territory with sTREM-1 but not sTREM-2 concentrations (Supplementary Table S2). Regarding the hemispheres, we did not show any differences. (Supplementary Table S3).

A total of twenty-three patients underwent therapy in the two hours after admission: fifteen received IV rtPA, four received only thrombectomy, and four received a combination of IV rtPA and thrombectomy. The other patients did not receive therapy.

The NIHSS at the admission had a mean of 9.84; one week after the ischemic event, the mean was 6.07, and the mRS after three months was 2.85.

Ten patients died within 3 months (Table 1).

**Figure 1:**
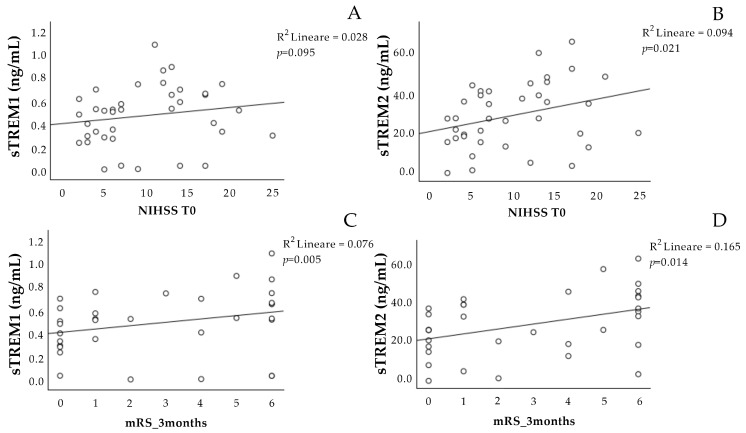
Correlation analysis of sTREM-1 and sTREM-2 at T0 with the stroke scales. Correlation between sTREM-1 (**A**,**C**) and sTREM-2 (**B**,**D**) concentration at admission (T0) with the NIHSS at T0 and the mRS scale after 3 months. NIHSS: National Institutes of Health Stroke Scale. mRS: Modified Rankin Scale. sTREM-1: soluble triggering receptor expressed on myeloid cell-1; sTREM-2: soluble triggering receptor expressed on myeloid cell-2.

The concentration of sTREM-1 at T0 was not associated with the NIHSS scale at T0 (Figure 1A), and it showed a significant positive association with the mRS scale at three months (Figure 1C).

The sTREM-2 at T0 was positively associated with the NIHSS scale at T0 (Figure 1B), as well as with the mRS scale at three months (Figure 1D).

**Figure 2:**
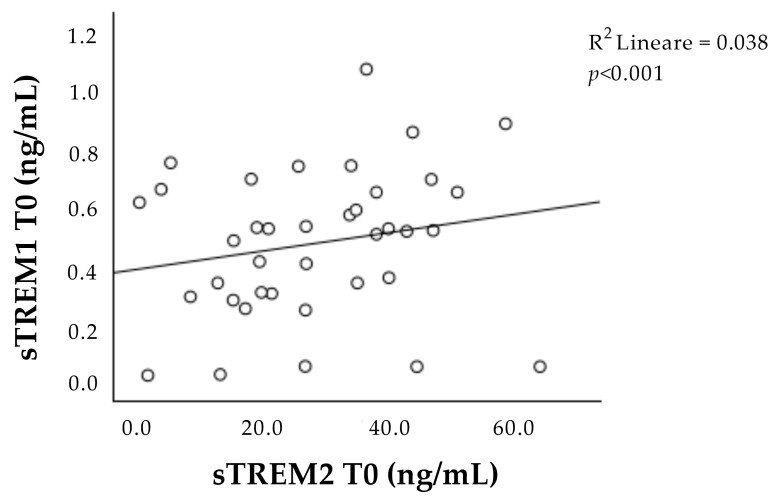
Correlation between sTREM-1 and sTREM-2 concentration at admission (T0). Positive correlation between sTREM-1 and sTREM-2 during the acute phase (T0). sTREM-1: soluble triggering receptor expressed on myeloid cell-1; sTREM-2: soluble triggering receptor expressed on myeloid cell-2.

Interestingly, the concentrations of sTREM-1 and sTREM-2 at T0 correlated with each other (Figure 2).

A general linear model adjusted for the age, sex, and subject that received or did not receive therapy (confounding factors) was performed to determine whether the plasma concentration of sTREM-1 and sTREM-2 differed during follow-up (T0 and TW) in a subgroup of patients (Table 2).

While sTREM-1 concentrations did not appear to vary significantly, the concentrations of sTREM-2 significantly decreased across the two follow-up times (Table 2).

The associations between the plasmatic concentrations of sTREM-1 and sTREM-2 with the mRS scale adjusted for the presence of therapy showed a significant positive association for both sTREMs (Table 3).

**Figure 3:**
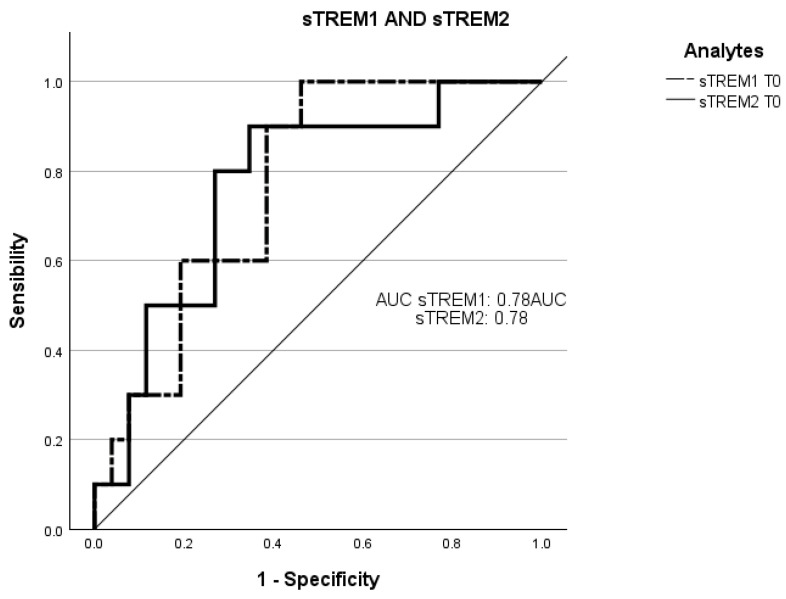
Receiver operating characteristic (ROC) curve analysis of sTREM-1 at T0 and sTREM-2 at T0 with death. The ROC curves of sTREM-1 and sTREM-2 concentration at admission point (T0) with the death of patients within three months. ROC, receiver operating characteristic; AUC, area under the curve; sTREM-1: soluble triggering receptor expressed on myeloid cell-1; sTREM-2: and soluble triggering receptor expressed on myeloid cell-2.

Receiver operating characteristic curve (ROC) analysis was employed to evaluate the ability of the plasmatic concentrations at T0 of sTREM-1 and sTREM-2 to predict negative prognosis (death of the patients in three months) (Figure 3).

The total area under the curve (AUC) of the concentration of sTREM-1 at T0 in predicting a worse prognosis was 78% (95% CI: 0.61–0.93; sensitivity: 83%; specificity: 35%).

The AUC of the concentration of sTREM-2 at T0 in predicting a worse prognosis was 78% (95% CI: 0.60–0.94; sensitivity: 83%; specificity: 35%).

## 3. Discussion

The main findings of this study were the association observed between the concentrations of sTREMs in the very early acute phase of AIS (within 4.5 h after arrival at the hospital) and the following decrease in the concentration of sTREM-2 during time, while the concentration of sTREM-1 remained similar.

Interestingly, the concentration of sTREM-2 on admission was correlated with both the NIHSS score at T0 and the mRS score at three months, while sTREM-1 only correlated with the mRS. Even after adjusting for the presence of therapy, the concentrations of both sTREMs on admission were associated with a worse prognostic outcome (mRS), and both sTREMs at admission were associated with mortality at three months by the means of ROC analyses.

These data are in line with our previous findings, showing that acute autonomic dysfunction was associated with a negative prognosis at three months. Similarly, the acute activation of intercellular and intersystemic pathways (e.g., specific subtypes of extracellular vesicles) has a poor prognostic value [19,20].

Neuroinflammation following stroke plays a crucial role in pathological processes that lead to adverse outcomes [21]. Even though the inflammatory cascade activates immediately after blood vessel occlusion, functional impairment develops days or weeks after the acute event [5].

This abnormal activation of neuroinflammation involves microglia activation, the infiltration of circulating leukocytes, the release of damage-associated molecular pattern molecules, and, finally, the production of pro-inflammatory cytokines and chemokines [14].

Several studies have shown that TREM-1 has a significant role in the activation of post-ischemic pro-inflammatory pathways both intracellularly (phosphorylation of DAP12, activation of the transcription factor NF-κB) and extracellularly (interaction with ligands, e.g., DAMP) [11,13,22]. In a mouse model in which ischemia was pharmacologically induced, it was observed that TREM-1 promotes the production of IL-1β, IL-18, IL-6, CXCL-2, MCP-1, and CXCL-1 in microglia [13].

Significant increase in serum TREM-1 levels after intracerebral hemorrhage (ICH) suggests its association with the inflammatory response, hemorrhage severity, and long-term functional prognosis. Consequently, sTREM-1 shows promising features as an inflammatory biomarker to assess ICH severity and predict early neurological function decline [23].

The results that we obtained on sTREM-1 concentration confirm what has been reported in the literature, that sTREM-1 has a role in enhancing stroke ischemic inflammation [11,22,23,24]. Indeed, in our patients, we found an association between sTREM-1 at T0 and the outcome three months after the acute event (mRS scale). This positive association with the prognosis at three months could be due to the fact that sTREM-1 rises in the acute phase, triggering such an elevated inflammatory state, with dose-dependent effects in the post-acute phase.

Furthermore, a recent study observed a correlation between the plasma levels of sTREM-1 and an increased risk of stroke at one month and six months after the acute event [24].

In contrast, we observed that the levels of sTREM-1 showed no variation in a small subset of patients between the acute phase (T0) and one week after the acute event (TW), presuming that the inflammatory state due to the initial insult remains unchanged over time. Vice versa, in our patients, we did not observe any correlation between sTREM-1 and the NIHSS scale at T0. A possible explanation might be due to the relatively small sample size of our patient group. In addition, other factors (stress, anxiety, comorbidities) might have altered acutely the outcome/status of the patients during ischemic stroke [25,26,27].

On the contrary, the role of sTREM-2 is not yet fully understood in AIS. The results of previous studies have not established whether this receptor has a pro-inflammatory role or an anti-inflammatory role. Numerous studies have focused on the role of TREM-2 as a microglial receptor, while few investigations have examined its soluble form in humans, likely due to the limited knowledge of the formation mechanisms and cellular implications of sTREM-2 [28,29,30,31].

The upregulation of TREM-2 in mice has been demonstrated to be neuroprotective, as evidenced by enhanced microglial polarization towards the M2 anti-inflammatory phenotype, while, by contrast, the reduction in TREM-2 levels in vivo exacerbated neuronal damage by increasing the release of pro-inflammatory cytokines [18]. In another study, the role of TREM-2 in microglial phagocytosis was examined in a mouse model of stroke, highlighting worsened post-stroke neurological outcomes due to microglial hyperactivation [15]. Hyperactivated microglia may lead to the release of various inflammation-related molecules and further compromise the blood–brain barrier function. This could allow inflammatory substances to pass into the brain, further amplifying the inflammatory response and contributing to neuronal damage [32,33,34].

The novelty of our study is a significant decrease during the iperacute phase in the plasma concentration levels of sTREM-2 between the two time points (T0 and TW) in a small subgroup of patients after adjusting for age, sex, and presence of therapy, which are all confounding factors.

Since ischemic stroke is a time-dependent condition [35], the analysis of the concentration of biomarkers within 4.5 h of the ischemic event could help untangle the biological mechanisms in the early phase of the event and give insight regarding the future prognostic course.

The reduction in sTREM-2 levels after one week may reflect the activation of the M1 microglial phenotype by sTREM-2. sTREM-2, which could enhance the recruitment of factors acting as compensatory mechanisms, triggered by an excess of pro-inflammatory cytokines released after the ischemic event during the acute phase. Subsequently, the activation of an appropriate inflammatory pathway (as evidenced by its activation at T0) could trigger the M2 microglial phenotype, which may secrete neurotrophic substances, remove necrotic or apoptotic neuronal debris, and attempt to try to resolve inflammation [18]. However, this heightened inflammation in the acute phase may disrupt the surrounding cellular and molecular environment, compromising the abilities of cells, including microglia, to perform their role in cellular debris removal and tissue homeostasis maintenance, leading to an ineffective resolution of the inflammatory state.

Furthermore, we observed a strong positive correlation between the concentration of both sTREM-1 and sTREM-2 during the acute phase of the ischemic event, suggesting the presence of some mutual interaction between TREM concentrations.

This mutual interaction seems to get lost after one week when the concentration of sTREM-2 decreases, while sTREM-1 remains the same, probably because prolonged TREM-1 signaling may activate negative feedback mechanisms that modulate TREM-2 [28,36,37].

This trend could explain why, in our cohort, we observed a correlation between the plasma concentration of sTREM-2 at the acute phase and in both the NIHSS score at the same time and three months after the acute event (mRS scale), while sTREM-1 only correlated with the mRS scale. The association between sTREM-2 concentration and the NIHSS score at admission may confirm the existence of a compensatory process that counteracts inflammation during the hyperacute phase after AIS. sTREM-2 is no longer able to cope with the high inflammatory state that remains persistent over time, which can lead to the chronicization of the inflammatory state and, consequently, may be associated with a negative outcome after three months.

These observations are further confirmed by the ROC curve in which the concentrations of both at T0, with mortality within 3 months, could predict an inauspicious prognosis.

In conclusion, we could hypothesize that the advantage of analyzing plasma concentrations of sTREM-1 and sTREM-2 lies in the fact that, being the soluble forms of receptors expressed on microglia cells, they could provide indications of what happens in the brain post-stroke. So, exploring these relationships, we sought to clarify the role of sTREMs as potential biomarkers that can provide useful indications regarding negative outcomes, even in the long term.

Our study has some limitations. Firstly, the patient cohort is relatively small, and different types of stroke are present. In addition, the blood samples after one week were only collected for a small subgroup of patients. However, all patients were recruited within 4.5 h from the onset of the event, ensuring accurate and homogeneous assessment times and consistent stroke pathophysiological phases. Moreover, sTREM-1 and sTREM-2 have not yet been well characterized in the very early phase of AIS at soluble levels and represent a novelty in neuroinflammation studies. Finally, we have the opportunity to analyze severity not only in the hyperacute phase but also after three months, in which long-term outcomes are poorly characterized.

In conclusion, future studies on the interaction between sTREM-1 and sTREM-2 in a larger patient cohort could define a potential promising pathway in the early diagnosis of stroke severity and a potential therapeutic target for long-term outcomes.

## 4. Materials and Methods

### 4.1. Study Design

For this study, patients admitted to the Emergency Department of Fondazione IRCCS Ca’ Granda, Ospedale Maggiore Policlinico in Milan from September 2016 to March 2018 who were diagnosed by a Computed Tomography scan (as the primary diagnostic method of screening) with AIS were enrolled.

Upon admission, demographic, anthropometric, clinical data, and biochemical analyses were also recorded.

Inclusion criteria required patients to exhibit new neurological symptoms within 4.5 h, persisting for at least 30 min. Eligible participants were adults over 18 years old with stable spontaneous sinus rhythm on the ECG at presentation.

Exclusion criteria comprised primary intracerebral hemorrhage, pre-existing neurological conditions, epilepsy at the onset of stroke symptoms, severe organ failure, active oncological conditions, mechanical ventilation, or refusal of consent.

Informed consent was obtained from all participants, and this study adhered to the principles of the Declaration of Helsinki.

All enrolled participants underwent diagnostic and therapeutic procedures in compliance with both the International and Internal Guidelines for the treatment of acute ischemic stroke, which have been in effect since 2016–2018 [38,39].

In adherence to the guidelines, intravenous thrombolytic therapy (r-tPA at 0.9 mg/kg; maximum total dose of 90 mg; 10% as IV bolus, and the remainder infused over 60 min) was planned for enrolled participants meeting specific criteria, excluding those with prior or current cerebral hemorrhage, bleeding disorders, and coagulopathy, prolonged activated partial thromboplastin time (aPTT), or those who had not received heparin in the previous 48 h [39,40]. Mechanical thrombectomy was planned for AIS participants with large-vessel occlusion who received intravenous r-tPA at admission, if eligible, and had symptom onset within 6 h or cases of contraindications to IV r-tPA [39,40,41].

The protocol received approval from the local Ethics Committee “Comitato Etico—Milano Area 2”, Milan, Italy (approval code 1443/2016).

### 4.2. Stroke Measurement

Stroke severity was gauged using the National Institutes of Health Stroke Scale (NIHSS), a comprehensive assessment tool consisting of 11 neurological items, with scores ranging from 0 to 42 (lower scores indicating less severe neurological impairment). The NIHSS evaluations were conducted at admission (T0) and one week later (Tw) [42], with an NIHSS score of ≥14 signifying severe conditions [42].

For mid-term outcome assessment and evaluation of residual functional disability, participants underwent a follow-up after 3 months, employing the Modified Rankin Scale (mRS), which ranges from 0 (no symptoms) to 6 (death) [43].

### 4.3. Plasma Samples Analysis

All patients underwent venous sampling for the evaluation of the plasmatic concentrations of triggering receptors expressed on myeloid cells 1 (sTREM-1) and triggering receptors expressed on myeloid cells 2 (sTREM-2). Fasting blood samples were collected in ethylenediaminetetraacetic acid (EDTA) tubes (7 mL), at admission within 4.5 h from the arrival at the hospital (T0) and after one week (Tw).

The blood samples were centrifuged at 1200× *g* for 15 min at room temperature to obtain platelet-free plasma. The resulting plasma was rapidly frozen and stored at −20 °C.

To quantify sTREM-1 and sTREM-2, the Human Simple Plex assays (ProteinSimple, Bio-Techne, Minneapolis, MN, USA) on an Ella device (Ella System, Bio-Techne, Minneapolis, MN, USA) were utilized.

Briefly, instrument calibration was carried out using the in-cartridge factory standard curve, and plasma samples were measured with dilution in a sample diluent as per the manufacturer’s instructions (ProteinSimple, CA, USA). Each sample was allocated to a single well, as the Simple Plex microfluidic platform automatically performs triplicate assays [44,45].

### 4.4. Statistical Analysis

The statistical analyses were carried out using IBM SPSS Statistics software (version 29, IBM Inc., Chicago, IL, USA).

The distribution of the demographic and clinical parameters was evaluated by a Kolmogorov–Smirnov test to assess possible deviations from the Gaussian model. Continuous data were expressed as the mean standard deviation (SD). Categorical data were presented as frequencies and percentages.

Linear general models were performed to compare the level of plasmatic concentration of sTREMs in the three time points and between the time points, adjusting by age, sex, and therapy.

Pearson’s correlations were performed for the parametric variable to explore the association between sTREM-1 and sTREM-2 concentrations (dependent variable) and the association with the severity of stroke by the NIHSS scale and the mRS scale.

Linear regression was performed to investigate the association of sTREM-1 and sTREM-2 with the mRS scale adjusted for the presence or absence of therapy.

Data of the plasmatic concentration of sTREM-1 and sTREM-2 were used in receiver operating characteristic (ROC) analysis to draw the ROC curve and to calculate the area under the curve (AUC) to observe the association of the level of sTREM-1 and sTREM-2 with the death of the patient.

Statistical significance was set at *p* < 0.05.

## Figures and Tables

**Table 1:** Demographic and clinical characteristics of the studied population (n = 38). Age, BMI, blood pressure, NIHSS (T0 and TW), and mRS scores are expressed as means (standard deviation), whereas all the other parameters are expressed as percentages.

Characteristics	Value
Age, years	72.9 (SD 14.7)
Gender (female)	15 (39.5%)
Smoker	9 (23.7%)
BMI kg/m^2^	24.7 (SD 3.55)
Hypertension	24 (63.2%)
History of atrial fibrillation	12 (31.6%)
Diabetes	6 (15.8%)
History of heart failure	5 (13.2%)
Blood pressure, mmHg:	
Systolic	160.08 (SD 24.04)
Diastolic	89.54 (SD 17.97)
Under therapy:	
IV rtPA	15 (39.5%)
Thrombectomy	4 (10.5%)
IV rtPA + thrombectomy	4 (10.5%)
No therapy	15 (39.5%)
Previous stroke or TIA	9 (23.7%)
TOAST classification:	
Atherothrombotic	5 (13.4%)
Cardioembolic	19 (50.0%)
Lacunar	2 (5.3%)
Undetermined etiology	12 (31.6%)
Hemispheric stroke:	
Right	18 (47.37%)
Left	18 (47.37%)
Bilateral	2 (5.26%)
Vascular territory:	
Anterior	31 (81.58%)
Posterior	7 (18.42%)
Three-month mortality (%)	10 (26.3%)
NIHSS T0 score	9.84 (SD 6.19)
NIHSS TW score	6.07 (SD 7.58)
mRS score	2.85 (SD 2.57)

Abbreviations: BMI: body mass index; TIA: transient ischemic attack; SD: standard deviation; NIHSS: National Institutes of Health Stroke Scale; TOAST: Trial of Org 10,172 in Acute Stroke Treatment (classification of stroke subtypes based on etiology as the main criterion); mRS: Modified Rankin Scale; rtPA: intravenous thrombolytic therapy.

**Table 2:** Concentrations of sTREM-1 and sTREM-2 during follow-up. Values are expressed as means (standard deviation). sTREM-1: soluble triggering receptor expressed on myeloid cell-1; sTREM-2: soluble triggering receptor expressed on myeloid cell-2.

	T0 (n = 38)	TW (n = 15)	*p*
sTREM-1 (ng/mL)	0.53 (0.19)	0.59 (0.27)	0.29
sTREM-2 (ng/mL)	31.51 (13.84)	21.25 (8.61)	0.003

**Table 3:** Linear regression analysis of sTREM-1 at T0 and sTREM-2 at T0 with the mRS at three months. Values are adjusted for the presence of therapy. sTREM-1: soluble triggering receptor expressed on myeloid cell-1; sTREM-2: soluble triggering receptor expressed on myeloid cell-2.

	R^2^	B	SE	*p*
sTREM-1 (ng/mL)	0.23	0.51	0.002	0.007
sTREM-2 (ng/mL)	0.20	0.42	0.000	0.01

SE: standard error for the unstandardized beta coefficent; R^2^: the coefficent of determination; B: the unstandardized betta coefficent.

## Data Availability

The data presented in this study are available upon request from the corresponding author.

## Supplementary Materials

The following supporting information can be downloaded at: https://www.mdpi.com/article/10.3390/ijms25147611/s1.
