# Supplementary material for: Soluble Triggering Receptors Expressed on Myeloid Cells (sTREM) in Acute Ischemic Stroke: A Potential Pathway of sTREM-1 and sTREM-2 Associated with Disease Severity
Source: Int J Mol Sci. 2024 Jul 11;25(14):7611. doi: 10.3390/ijms25147611 (PMC11277504; doi:10.3390/ijms25147611)
Supplement: Supplementary file 1 [file ijms-25-07611-s001.zip › ijms-3024019-supplementary.pdf]

Supplementary materials

Table S1 (A,B,C,D,E,F). Correlation analysis between stroke's type and TREMs concentration

A

	Atherothrombotic (n=5)	Cardioembolic (n=19)	p
sTREM-1 T0 (ng/mL)	0.49 (0.14)	0.55 (0.22)	0.55
sTREM-2 T0 (ng/mL)	30.72 (17.66)	32.63 (15.29)	0.81

B

	Atherothrombotic (n=5)	Lacunar (n=2)	p
sTREM-1 T0 (ng/mL)	0.49 (0.14)	0.55 (0.05)	0.59
sTREM-2 T0 (ng/mL)	30.72 (17.66)	38.95 (5.76)	0.56

C

	Atherothrombotic (n=5)	Undetermined etiology (n=12)	p
sTREM-1 T0 (ng/mL)	0.49 (0.14)	0.49 (0.20)	0.97
sTREM-2 T0 (ng/mL)	30.72 (17.66)	28.81 (11.16)	0.78

D

	Cardioembolic (n=19)	Undetermined etiology (n=12)	p
sTREM-1 T0 (ng/mL)	0.55 (0.22)	0.49 (0.20)	0.40
sTREM-2 T0 (ng/mL)	32.63 (15.29)	28.81 (11.15)	0.46

E

	Cardioembolic (n=19)	Lacunar (n=2)	p
sTREM-1 T0 (ng/mL)	0.55 (0.22)	0.55 (0.05)	0.99
sTREM-2 T0 (ng/mL)	32.63 (15.29)	38.95 (5.76)	0.58

F

	Lacunar (n=2)	Undetermined etiology (n=12)	p
sTREM-1 T0 (ng/mL)	0.55 (0.05)	0.49 (0.20)	0.66
sTREM-2 T0 (ng/mL)	38.95 (5.76)	28.81 (11.15)	0.24

Table S1 (A,B,C,D,E,F). Values are expressed as mean (standard deviation). Correlation analysis between the four different type of stroke and both sTREM concentrations.

TREM-1: Soluble Triggering receptor expressed on myeloid cell-1; TREM-2: and soluble Triggering receptor expressed on myeloid cell-2.

Table S2. Correlation analysis between vascular territory and TREMs concentration.

	Anterior (n=29)	Posterior (n=9)	p
sTREM-1 T0 (ng/mL)	0.56 (0.19)	0.38 (0.13)	**0.03**
sTREM-2 T0 (ng/mL)	32.65 (14.46)	26.46 (9.91)	0.29

Table S2. Values are expressed as mean (standard deviation). TREM-1: Soluble Triggering receptor expressed on myeloid cell-1; TREM-2: and soluble Triggering receptor expressed on myeloid cell-2.

Table S3. Correlation analysis between hemispheres and TREMs concentration.

A

	Unilateral (n=36)	Bilateral (n=2)	p
sTREM-1 T0 (ng/mL)	0.53 (0.20)	0.33 (0.10)	0.16
sTREM-2 T0 (ng/mL)	32.14 (13.89)	20.06 (9.73)	0.23

B

	hemisphere left (n=18)	hemisphere right (n=18)	p
sTREM-1 T0 (ng/mL)	0.50 (0.19)	0.57 (0.19)	0.27
sTREM-2 T0 (ng/mL)	32.30 (13.95)	31.99 (14.15)	0.95

Table S3. Values are expressed as mean (standard deviation). TREM-1: Soluble Triggering receptor expressed on myeloid cell-1; TREM-2: and soluble Triggering receptor expressed on myeloid cell-2.
